# Development and implementation of a mini-Clinical Evaluation Exercise (mini-CEX) program to assess the clinical competencies of internal medicine residents: from faculty development to curriculum evaluation

**DOI:** 10.1186/1472-6920-13-31

**Published:** 2013-02-26

**Authors:** Kuo-Chen Liao, Shou-Jin Pu, Maw-Sen Liu, Chih-Wei Yang, Han-Pin Kuo

**Affiliations:** 1 Division of General Medicine and Geriatrics, Department of Internal Medicine, Chang Gung Memorial Hospital, Chang Gung University, College of Medicine, 5 Fusing street, Gueishan, 333, Taoyuan, Taiwan; 2 Division of Nephrology, Department of Internal Medicine, Chang Gung Memorial Hospital, Chang Gung University, College of Medicine, Taoyuan, Taiwan; 3 Division of Thoracic Medicine, Department of Internal Medicine, Chang Gung Memorial Hospital, Chang Gung University, College of Medicine, Taoyuan, Taiwan

**Keywords:** Mini-CEX, Mini clinical evaluation exercise, Faculty development

## Abstract

**Background:**

The mini-CEX is a valid and reliable method to assess the clinical competencies of trainees. Its data could be useful for educators to redesign curriculum as a process of quality improvement. The aim of this study was to evaluate a mini-CEX assessment program in our internal medicine residency training. We investigated the impact of mini-CEX workshops as a faculty development program on the acquisition of cognitive knowledge and the difference of practice behaviors among faculty members used the mini-CEX to assess residents’ performance at work.

**Methods:**

We designed an observational, two-phase study. In the faculty development program, we started a mini-CEX workshop for trainers in 2010, and the short-term outcome of the program was evaluated by comparing the pretest and posttest results to demonstrate the improvement in cognitive knowledge on mini-CEX. From September 2010 to August 2011, we implemented a monthly mini-CEX assessment program in our internal medicine residency training. The data of these mini-CEX assessment forms were collected and analyzed.

**Results:**

In the group of 49 mini-CEX workshop attendees, there was a statistically significant improvement in cognitive knowledge by comparing the pretest and posttest results (67.35 ± 15.25 versus 81.22 ± 10.34, *p* < 0.001). Among the 863 clinical encounters of mini-CEX, which involved 97 residents and 139 evaluators, 229 (26.5%), 326 (37.8%), and 308 (35.7%) evaluations were completed by the first-year, second-year, and third- year residents separately. We found a statistically significant interaction between level of training and score in dimensions of mini-CEX. The scores in all dimensions measured were better for senior residents. Participation in mini-CEX workshops as a faculty development program strengthened the adherence of trainers to the principles of mini-CEX as a formative assessment in regard to provision of feedback. However, a deficiency in engaging residents’ reflection was found.

**Conclusions:**

Faculty development is a prerequisite to train evaluators in order to implement a successful mini-CEX assessment program. We demonstrated the effectiveness of our mini-CEX workshops in terms of knowledge acquisition and enhancement of giving feedback when the faculty members used the tool. Further programs on providing effective feedback should be conducted to increase the impact of the mini-CEX as a formative assessment.

## Background

Training competent physicians to improve the quality of health care and to meet societal expectations are the primary goals of current medical education [1]. It has been asserted that educators should design a competency-based training program guided by the principles and structures of outcome-based education [2]. In 1998, the Accreditation Council of Graduate Medical Education (ACGME) began an initiative, called the Outcome Project, which fostered residency training with a focus on development and assessment of the six competencies, including medical knowledge, patient care, interpersonal and communication skills, systems-based practice, professionalism, and practice-based learning and improvement [3]. Among the assessment tools targeted on various competencies evolving for years, the direct observation at workplace has played an important role in the process of these educational reforms [4,5]. In addition to serving as the guidance for feedback on learners’ performance, the results of various assessments could be useful for educators to modify their training programs as a process of quality improvement [6,7].

The American Board of Internal Medicine (ABIM) developed a quality assessment tool, the mini-Clinical Evaluation Exercise (mini-CEX), which has been applied internationally to assess the clinical competencies of trainees at different subspecialties in a wide variety of clinical settings [8-11]. For each mini-CEX encounter, one evaluator observes the resident conduct a focused interview, physical examination, or therapeutic counseling at a selected workplace, followed by provision of immediate feedback and completion of the rating form. With its feature to provide feedback following a clinical assessment, the mini-CEX also serves as a formative method to guide trainees’ professional development. Previous research on the mini-CEX focused on its validity, reliability, and feasibility to assess the clinical skills of residents, and the educational impact of effective feedback to foster their future learning and improvement [12-14].

Since 2006, the Taiwan Association of Medical Education (TAME) facilitated several medical centers to establish the “General Medicine Training Demonstration Center” with support from the Department of Health, aiming to promote the postgraduate training and provide a holistic patient-centered quality care in Taiwan. An additional project named “The Faculty Development Program for Postgraduate Training” started in 2009 with objectives to equip the clinical teachers with the capabilities of teaching and assessing the ACGME six competencies of trainees [15]. Our hospital was one of the certified institutions to implement both “General Medicine Training Demonstration Center” and “The Faculty Development Program for Postgraduate Training” projects. We conducted a two-hour mini-CEX workshop to train these clinical teachers to acquire the cognitive knowledge on mini-CEX and its clinical applications. In September 2010, we proposed a monthly mini-CEX assessment program to the Department of Internal Medicine and each resident was required to be assessed during the course of training.

The aim of this study was to evaluate a mini-CEX assessment program in our internal medicine residency training. We specifically investigated the impact of mini-CEX workshops as a faculty development program on the acquisition of cognitive knowledge and the difference of practice behaviors among evaluators used the mini-CEX to assess residents’ performance at work.

## Methods

### Study design

We designed this observational, two-phase study to evaluate a mini-CEX assessment program in our internal medicine residency training by reporting on the outcomes of mini-CEX workshops as a faculty development program and identifying potential opportunities for curricular improvement. Ethical approval was obtained from the Institutional Review Board of Chang Gung Memorial Hospital.

### The faculty development program

We organized a two-hour mini-CEX workshop as one of the assessment curricula within the project of Faculty Development Program of Postgraduate Training. The workshop comprising a pretest, mini-lecture, video-clip rating exercise, small group discussion, and a posttest. A multiple-choice-question test on mini-CEX was designed as the pretest and posttest (Additional file 1). Its contents included the cognitive knowledge on assessment of clinical competence, the application of the mini-CEX as a workplace-based assessment tool, and the principles of providing effective feedback. Improvement in cognitive knowledge was assessed by comparing the results between the pretest and posttest. A total of 49 participants attended the mini-CEX workshops were enrolled.

### The monthly mini-CEX assessment program

In September 2010, we started a monthly mini-CEX assessment program for internal medicine residents. A modified ABIM mini-CEX assessment form was adopted, including assessing six dimensions of performance and one global rating on overall clinical competence. The six dimensions of performance were medical interviewing skills, physical examination skills, counseling skills, clinical judgment, humanistic qualities / professionalism, organization and efficiency. We specifically listed a descriptor of each dimension in the mini-CEX assessment form following each rating for reference. Additionally, we divided the space for comment into two categories, resident’s reflection and evaluator’s feedback respectively. As part of the implementation of the mini-CEX program, we provided a grand round didactic lecture titled “ The mini-CEX as a workplace-based assessment” for all the residents and faculty members of internal medicine, addressing the issues of in-training evaluation and the procedural aspects of the mini-CEX as a formative assessment. The faculty members were asked to assess residents’ performance monthly and handed the completed mini-CEX assessment forms to the office of Internal Medicine for statistical analysis.

### Feedback analysis

To exam the quality of feedback, two authors (K.C.L. and S.J.P.) reviewed the assessment forms gathered and classified the contents of feedback and reflection. We found that several contents of the written feedback were not easy to be categorized into the dimensions measured by the mini-CEX. That is, most evaluators provided and documented comments in a more customary way rather than organized feedback according to the order of the competencies measured on the mini-CEX form. Similar findings have been described in literature [16]. In order to demonstrate authenticity and avoid misinterpretation, we decided to categorize the contents of written feedback into five specific aspects after discussion, including history taking, physical examination, counseling and communication skills, attitudes/professionalism, and clinical reasoning. Previous literature also addressed that engagement of resident’s reflection by evaluators was regarded as an indicator of effective feedback [17]. The contents of reflection were conventionally grouped into medical knowledge, clinical skills, and attitude/professionalism (Additional file 2).

### Statistical analysis

The continuous variables were expressed as means and standard deviations and compared using Student’s *t*-tests. The categorical variables were summarized as proportions and compared using Chi-square tests or Fisher’s exact tests. A *p*-value of less than 0.05 was taken as statistically significant. A repeated measures analysis of variance (RM-ANOVA) was conducted to evaluate the interaction between the score in dimensions of mini-CEX (within-subjects factor) and level of training (between-subjects factor). All data were analyzed using the Statistical Package for the Social Sciences (SPSS) 17.0 for Windows.(Chicago, IL, USA).

## Results

### The mini-CEX workshops as a faculty development program

In 2010, a total of 67 trainers of different subspecialties joined our program. Table 1 shows descriptive statistics for 49 trainers attended the mini-CEX workshops. There were 5 (10.2%) female and 44 (89.8%) male trainers, including 17 internists, 13 surgeons, 6 obstetricians/gynecologists, 3 pediatricians, and 10 of other specialties (family medicine, emergency medicine, and psychiatry). The mean scores of pretest and posttest were 67.35 ± 15.25 and 81.22 ± 10.34, respectively. Improvement in cognitive knowledge was statistically significant ( *p* < 0.001 ).

**Table 1:** Descriptive statistics for 49 trainers attended the mini-CEX workshops in 2010

**Variables**	**Gender**		**Number**
**Gender**	**Male**	**Female**	**Total (%)**
Specialty			
Internal Medicine	13	4	17 (34.7)
Surgery	13	0	13 (26.5)
Obstetrics/Gynecology	5	1	6 (12.2)
Pediatrics	3	0	3 (6.1)
Others : Psychiatry, Family Medicine, Emergency Medicine	10	0	10 (20.4)
Total (%)	44 (89.8)	5 (10.2)	49 (100)

### The monthly mini-CEX assessment program

From September 2010 to August 2011, the data of monthly mini-CEX of internal medicine residents were collected and analyzed, which composed of 863 clinical encounters involving 97 residents and 139 evaluators. Every resident received a mean number of 8.9 mini-CEX assessments (standard deviation 4.2; range 1-20), while every evaluator completed a mean number of 6.2 assessment forms (standard deviation 6.2; range 1-38). Among these 863 clinical encounters, 229 (26.5%) assessment forms were completed by the first-year residents, 326 (37.8%) by the second-year residents, and 308 (35.7%) by the third-year residents, while 402 (46.6%) encounters were assessed by chief residents and 461 (53.4%) encounters assessed by attending physicians. The type of visit was mainly new patient interview (45.8%), and most encounters occurred at inpatient wards (92.9%) and were regarded as moderate in complexity (78.7%) for the mini-CEX assessment. These encounters were focused chiefly on clinical judgment (75.4%), diagnosis and treatment (71.6%), and data collection (54.1%). Both residents and evaluators were satisfied with the mini-CEX assessment. The descriptive data are shown in Table 2. Additionally, in Table 3, we found a statistically significant interaction between the level of training and score in dimensions of mini-CEX. A hypothesis that the level of training would affect performance was significant; the scores in all dimensions of mini-CEX measured were better for senior residents.

**Table 2:** Descriptive statistics in the mini-CEX assessment forms collected from September 2010 to August 2011

**Variable**	**Category**	**Number (%)**
Trainee	Total	863(100)
	R1	229(26.5)
	R2	326(37.8)
	R3	308(35.7)
Evaluator	CR/Fellow	402(46.6)
	Attending physician	461(53.4)
Type of visit	New patient	395(45.8)
	Follow up patient	273(31.6)
	Missing	195(22.6)
Setting	Ambulatory	32(3.7)
	Inpatient	796(92.2)
	Missing	35(4.1)
Complexity	Low	21(2.4)
	Moderate	679(78.7)
	High	92(10.7)
	Missing	71(8.2)
Focus of evaluation	Data collection	467(54.1)
	Clinical judgment	651(75.4)
	Diagnosis/treatment	618(71.6)
	Counseling/education	373(43.2)
	Missing	124(14.4)
Satisfaction	By resident	Mean ± SD
	Total	7.96(0.9)
	R1	7.84(0.9)
	R2	7.97(0.9)
	R3	8.04(0.9)
Satisfaction	By evaluator	
	Total	7.98(0.8)
	CR /Fellow	7.87(0.9)
	Attending physician (workshop attendees)	8.07(0.7)
	Attending physician (non- workshop attendees)	8.08(0.7)
Observation time	R1	16.88(8.7)
	R2	19.18(10.5)
	R3	20.29(10.9)
Feedback time	R1	12.88(9.5)
	R2	14.99(11.9)
	R3	13.64(8.7)

SD, standard deviation; R1: first- year resident; R2: second-year resident; R3: third-year resident; CR: chief resident.

**Table 3:** Evaluation of the interaction between level of training and score in dimensions of mini-CEX by repeated measures analysis of variance (RM-ANOVA)

**Source**	**Sum of squares**	**d.f.**	**Mean square**	***F*-value**	***p*-value**
Level of training	113.270	2	56.635	13.382*	.000
Score in dimensions of mini-CEX	33.810	5.566	6.075	29.153*	.000
Level of training x Dimensions of mini-CEX	8.161	11.132	0.733	3.518*	.000
Error					
Between subjects	3457.727	817	4.232		
Residual Error	947.520	4547.337	0.208		

*the level of significance was set at 0.05; d.f.: degree of freedom.

We reviewed the feedback of the collected mini-CEX assessment forms and studied their characteristics. Among the 863 mini-CEX encounters, 74.9% were regarded as providing proper feedback. In these mini-CEX assessment forms with feedback, 61.2% evaluators engaged resident’s reflection, which mainly addressed on medical knowledge (55.3%) and clinical skills (79.2%), but less frequently on attitudes/ professionalism (22.7%) as shown in Table 4.

**Table 4:** Categories of feedback and reflection in the mini-CEX assessment

**Mini-CEX encounters**	**n**	**%**
Total	863	100
Without feedback	217	25.1
With feedback	646	74.9
Categories of feedback		
History taking	147	22.8
Physical examination	182	28.2
Counseling / communication skills	163	25.2
Attitudes / Professionalism	331	51.2
Clinical reasoning	262	40.6
Without engagement of resident’s reflection	335	38.8
With engagement of resident’s reflection	528	61.2
Categories of reflection		
Medical Knowledge	290	55.3
Clinical skills	418	79.2
Attitudes / Professionalism	120	22.7

In Table 5, we compared the provision of feedback, engagement of resident’s reflection, satisfaction with mini-CEX, and total time spent on mini-CEX between attending physician evaluators with and without participation in mini-CEX workshops as a faculty development program. The attendees of mini-CEX workshops tended to provide feedback to residents (*p* = 0.003), but less frequently engaged resident’s reflection (*p* = 0.045). We found no differences in satisfaction with mini-CEX and the total time spent either on observation or feedback between the two groups.

**Table 5:** Comparison of mini-CEX encounters assessed by different evaluators

**Group**	**A**	**B**	***p *value**
**Number (%)**	**151(32.8)**	**310(67.2)**	
Providing feedback	123(81.5)	211(68.1)	0.003
Engaging resident reflection	62(41.1)	158(51.0)	0.046
Evaluator Satisfaction	8.23 ± 0.66	8.14 ± 0.70	0.944
Resident Satisfaction	8.24 ± 0.88	8.17 ± 0.73	0.105
Observation time	21.34 ± 12.75	17.64 ± 11.82	0.085
Feedback time	12.97 ± 6.96	15.46 ± 10.90	0.054

Group A: Attendees of mini-CEX workshops.

Group B: Non-Attendees of mini-CEX workshops.

## Discussion

To increase the impact of the mini-CEX as a formative assessment, a faculty development program or rater training program plays an important role. A variety of faculty development programs have been designed and implemented successfully in worldwide institutions [18-20]. It is also the obligations of educators to choose proper assessment methods to evaluate the outcomes of education linking with the objectives and goals of their curricula. In our study, even though we successfully demonstrated the short-term outcome of mini-CEX workshops as a faculty development program by an improvement in cognitive knowledge, we still questioned the sustainability of these gains and effects on trainers’ practice behaviors of teaching at workplaces. Analyzing the collected data of mini-CEX, we found that the evaluators with participation in the faculty development program tended to adhere to the principles of mini-CEX as a formative assessment by providing feedback to trainees more frequently. These results indirectly demonstrated the sustained impact of our faculty development program. However, there was a deficiency in engaging trainee’s reflection found in the group of workshop attendees. According to Archer’s model for effective feedback [21], which addressed self-monitoring (reflection on action) supported by external feedback and linkage with personal goals (action plan) in a coherent process, the result in our study points out future improvement for faculty development program through the process of curriculum evaluation.

We achieved the goal to assess residents’ performance confronting with patients of different complexities under various circumstances in our monthly mini-CEX assessment program. These mini-CEX results were regarded as components of a continuous learning curve for each resident and offered a clear guidance for both residents and educators to bring about personal and curricular improvement. In the meanwhile, the assessment process provided an interaction between residents and evaluators, either in communicating contents of feedback and future improvement strategies with each other, or in evaluators’ role-modeling approaches to patients during or after the exercise as a teaching method [22]. A previous qualitative study showed that the residents perceived the mini-CEX as anxiety-provoking because of its dual roles of assessment and education [23]. To implement a faculty development and longitudinal assessment program throughout the continuum of education, recruiting both residents and educators could be a solution to relieve mutual tensions and assume a constructive attitude toward clinical assessments [14].

Our study also demonstrated that the mini-CEX was a feasible tool to evaluate professional development of residents since level of training was significantly attributed to higher scores in all dimensions measured. When used alone, the mini-CEX may be insufficient to reflect trainees’ mastery of each competency. Previous studies showed the validity of the mini-CEX was supported by the strong correlations in scores between the mini-CEX and other assessments [5,24]. Residents’ performance mature gradually at each level and their learning should be facilitated by means of providing effective feedback at each teachable moment. Compared with the results in feedback analysis, attitude/professionalism was the commonest category of feedback; on the contrary, this was the least frequent category of resident’s reflection in our study. Formative assessment as the mini-CEX has its strength in real-time observation on a trainee’s attitude toward patients during encounters at different workplaces. To maximize the effect of feedback, we must nurture trainee reflection-in-action rather than a trainer-driven, but a two-way process in which trainers provide comments and at the same time encourage trainees to self-reflect on their performance [21]. The cultivation of professionalism in residency program requires organizational approaches since the social and educational environments of training institutions have a profound influence on the hidden curriculum. It has been challenging to demonstrate this competency learnt and internalized by residents even though there are evolving assessment tools specifically focusing on professionalism [25-27]. The mini-CEX was a practical tool for evaluators to observe contexts of professionalism expressed in a resident’s interaction with patients. Promoting a culture of professionalism and providing opportunities for self-reflection must be built into our current program to foster both cognitive and behavioral changes [28].

Although more of the participants attending our mini-CEX workshops provided recommendations as feedback, those without participation spent more time giving feedback and engaged residents’ reflection more often. Further qualitative studies should be conducted to investigate this contradictory finding, such as the perceptions from evaluators and trainees, specificity of feedback contents, and process of delivering feedback. Provision of feedback from the evaluators to residents after observing resident-patient interaction is a complex and dynamic process and is influenced by many variables, including faculty members’ tensions in balancing positive and negative feedback, their own perceived self-efficacy, their perceptions of the resident’s insight, skill, and potential, the faculty member-resident relationship and contextual factors [16]. Interventions like workshops on provision of effective feedback or modification of the assessment instrument could be considered in future programs to facilitate interactive and high-quality feedback in the mini-CEX [17,29].

Our study has several limitations. First, this was a single specialty and institution study and the results could not be generalizable to residents in other settings or contexts. The institutional environment is an important determinant for implementing a long-term formative assessment program and developing support of a feedback culture. Second, the retrospective analysis on the written feedback might preclude the actual feedback contents and also lead to underestimation of feedback quantity. An approach of video-taped sessions in the mini-CEX might be an alternative method to overcome this limitation. Nevertheless, videotaping is both time and labor-consuming and even difficult to conduct in a long-term continuous assessment program. Third, when considering workshop effectiveness, the faculty should be evaluated using models that evaluate their ability as raters, such as generalizability analysis or variance components analysis. These statistical models would be helpful to determine how much variance in scores is due to the raters. The multiple-choice-question pretest/ posttest we used in the study could only demonstrate the short-term outcome of mini-CEX workshops in knowledge acquisition. Finally, a substantial amount of missing data in each mini-CEX assessment form would bias the interpretation of our results.

## Conclusion

We demonstrated the outcomes of mini-CEX workshops as a faculty development program by providing the evidence of improvement in cognitive knowledge and its sustained impact on the practice behaviors of workshop attendees when they used mini-CEX to assess residents’ performance in terms of providing feedback. Using the data from the mini-CEX assessment program, we also demonstrated the feasibility of this instrument to monitor the professional development of internal medicine residents. Future faculty development program specifically on giving effective feedback should be provided to facilitate residents’ learning as a process of quality improvement.

## Consent

This study was approved by the ethical review board of Chang Gung Memorial Hospital. The faculty members and residents of internal medicine were informed of the purpose of the study and assured of confidentiality. Written informed consents were obtained from the participants enrolled for publication of this report.

## Competing interests

The authors declare that they have no competing interests.

## Authors’ contributions

KCL designed the mini-CEX written test, conducted the mini-CEX workshop, and served as the mini-CEX grand round lecturer. KCL and SJP developed the design of this study and were responsible for data collection, statistical analysis, and manuscript drafting. MSL, CWY, HPK contributed to the interpretation of data and feedback on the manuscript. All authors read and approved the final manuscript.

## Pre-publication history

The pre-publication history for this paper can be accessed here:

http://www.biomedcentral.com/1472-6920/13/31/prepub

## Supplementary Material

Additional file 1 Mini-CEX pre/posttest. Click here for file

Additional file 2 Categorization of evaluator’s feedback in feedback analysis. Click here for file
