# Supplementary material for: Development and implementation of a mini-Clinical Evaluation Exercise (mini-CEX) program to assess the clinical competencies of internal medicine residents: from faculty development to curriculum evaluation
Source: BMC Med Educ. 2013 Feb 26;13:31. doi: 10.1186/1472-6920-13-31 (PMC3599226; doi:10.1186/1472-6920-13-31)
Supplement: Additional file 1 — Mini-CEX pre/posttest. [file 1472-6920-13-31-S1.doc]

# Mini-CEX pre/posttest

1. **What is Mini-CEX ( Mini-Clinical Evaluation Exercise )?**
2. An assessment tool
3. An educational method
4. Focusing on direct observation of student’s performance
5. Giving immediate feedback
6. All of above
7. **In Miller’s Classification for methods of Assessment, Mini-CEX falls into which level of assessment?**
8. Does
9. Shows how
10. Knows how
11. Knows
12. **Mini-CEX was developed because of following reasons, except .**
13. To know what students have learned
14. To observe how students performed in clinical settings
15. To correct student’s bad behaviors in front of the patient
16. To give feedback toward students’ performance
17. To facilitate students’ improvement in the future
18. **Before starting the Mini-CEX, we should discuss with the students on the focus including .**
19. Data gathering
20. Diagnosis
21. Therapy
22. Counseling
23. At least one of above
24. **Mini-CEX was designed to be done within .**
25. 5 min
26. 15 min
27. 30 min
28. 60 min
29. 2 hour
30. **Mini-CEX can be scheduled in the following clinical settings .**
31. Ambulatory care
32. Inpatient care
33. Emergency department
34. Preoperative consultation
35. All of above
36. **What are observed ( Student’s performance ) in Mini-CEX ?**
37. Medical interviewing skills and physical examination skills
38. Humanistic qualities and professionalism
39. Clinical judgment
40. Counseling skills and Organization/efficiency
41. All of above
42. **What are the characteristics of giving effective feedback, except .**
43. Immediate and be specific
44. Focused on behaviors and personality
45. Achieving mutual agreement
46. Discussing on future learning plan
47. Providing chance for reassessment
48. **Which factors will potentially affect Mini-CEX observation ratings?**
49. Student’s background
50. No clear learning objectives
51. Complexity of the clinical case
52. Rater without Mini-CEX training
53. All of above
54. **What are the limitations of Mini-CEX, except .**
55. Standardized patient needed
56. Teacher’s rating bias
57. Lack of time
58. Hallo effect across the competencies
59. All of above
