# Supplementary material for: Development and implementation of a mini-Clinical Evaluation Exercise (mini-CEX) program to assess the clinical competencies of internal medicine residents: from faculty development to curriculum evaluation
Source: BMC Med Educ. 2013 Feb 26;13:31. doi: 10.1186/1472-6920-13-31 (PMC3599226; doi:10.1186/1472-6920-13-31)
Supplement: Additional file 2 — Categorization of evaluator’s feedback in feedback analysis. [file 1472-6920-13-31-S2.docx]

Additional file 2

Categorization of evaluator’s feedback in feedback analysis.

| Category | Example |
| --- | --- |
| History taking | |
|  | The resident seemed unfamiliar with how to initiate a history taking process and relate it to patient’s chief complaint… |
|  | …to improve interviewing skills for AIDS patients. |
| Physical examination | |
|  | …the resident could perform a focused physical examination to assist his clinical judgment. |
|  | The trainee efficiently demonstrated physical examination skills for a patient with secondary pneumothorax. |
| Counseling and communication skills | |
|  | …in the beginning, the resident should not forget to introduce himself to the patient and family….. |
|  | The resident could clearly explain the indications for operation (Psoas muscle abscess) to the patient after consulting the orthopedic surgeon. |
| Attitudes/ professionalism | |
|  | The trainee demonstrated his respect for the patient during the encounter and built up a good doctor-patient relationship. |
|  | …the trainee should cultivate and show his empathy toward the patient’s illness. |
| Clinical reasoning | |
|  | The resident could make a reasonable judgment promptly from the clinical symptoms and signs of a patient with UGI bleeding… |
|  | … put the information from history taking and physical examination together to generate a reasonable diagnosis in this patient ( pneumothorax ) |

Categorization of resident’s reflection in feedback analysis.

| Category | Example |
| --- | --- |
| Medical knowledge | |
|  | This cirrhotic patient has many contradictory conditions, like avoidance of constipation to prevent hepatic encephalopathy, but laxative overuse might lead to diarrhea and even hemorrhoid bleeding. |
|  | This rotation improved my knowledge on evaluation and diagnosis of chronic kidney disease… |
| Clinical skills | |
|  | I have to improve my communication skills, especially when I explain the patient’s condition and prognosis at work. |
|  | …in the whole care process, I should improve my skills to educate the patient and family about confronting with such a debilitating condition (chronic kidney disease) and address the importance of medication safety issues. |
| Attitude/professionalism | |
|  | It’s quite challenging and takes me patience and time to communicate with the family members because they are anxious and worried about the rapid changes of the patient’s condition. I have to learn how to deal with similar situations. |
|  | …need to strengthen my compassion for the patient’s predicament resulting from his illness. (chronic obstructive pulmonary disease) |
